# Comparative morpho-anatomical study on *Leptadenia pyrotechnica* (Apocynaceae) growing in the hyper-arid and arid habitats of Saudi Arabia

**DOI:** 10.7717/peerj.15320

**Published:** 2023-05-22

**Authors:** Najla A. Al Shaye

**Affiliations:** Department of Biology, College of Science, Princess Nourah bint Abdulrahman University, Riyadh, Saudi Arabia

**Keywords:** *Leptadenia pyrotechnica*, Morpho-anatomical adaptions, Hyper-arid, Arid habitats, Sand dunes, Empty Quarter, Jazan

## Abstract

The use of wild plants is considered to be an eco-friendly and promising natural resource. In sandy desert habitats, *Leptadenia pyrotechnica* flourishes as a xerophytic shrub with high biomass production. As a dominant shrub in the arid sand dune habitats of Saudi Arabia, *Leptadenia pyrotechnica* (Forssk.) Decne (Asclepiadaceae) is one of the most common xerophytes; the many medicinal uses of this plant include treating allergies, productive coughs, abortions, diabetes, stomach disorders, fevers, kidney disorders, and stones. In such a distribution, morpho-anatomical characteristics, among other adaptive traits, play an important role. This study aims to characterize some morpho-anatomical adaptations of *L. pyrotechnica* in two stressful habitats: the hyper-arid inland sand dunes of the Empty Quarter (EQ) and the arid coastal sand dunes of Jazan. A morpho-anatomical analysis of plant stems and roots from both habitats was conducted using light microscopy (LM) and scanning electron microscopy (SEM). The outcomes indicated similar characteristics, a low surface-to-volume ratio (S/V), a thin boundary layer (δ^bl^), an epidermis with many layers of hypodermis, bundles of sclerenchymatous cells around vascular tissue, and storage starch grains in ray parenchyma between xylem conduits. On the other hand, the *L. pyrotechnica* stem from the hyper-arid habitat of the Empty Quarter revealed more encrypted stomata, more elongated palisade cells, less calcium oxalate crystals with low Ca percentage, and a high vulnerability index of xylem vessels compared with the same traits of the stem from the Jazan coastal sand dunes habitat. Roots of *L. pyrotechnica* from both habitats revealed similar features of general anatomy. However, differences in specific anatomical traits were observed, especially in xylem vessel characters. The vulnerability index of root xylem vessels from the Empty Quarter habitat was more than that from the Jazan coastal sand dunes habitat. Furthermore, vestured bordered pits in root xylem walls were more abundant in the Empty Quarter habitat than in the Jazan coastal sand dunes habitat. As a result, these characteristics in the morpho-anatomy of *L. pyrotechnica* from both habitats provide practical adaptations to highly stressful conditions, along with specific anatomical traits relating to each habitat.

## Introduction

A relatively high species diversity characterizes southwest Saudi Arabia; several geomorphological characteristics contribute to this diversity, such as islands, sand dunes, sandy plains, low rocky hills, and high mountains (Masrahi, 2012). The floristic explorations carried out in this part of Saudi Arabia in recent years have led to the discovery of many new taxa (Al-Turki, Omer & Ghafoor, 2001; Al-Turki, Al-Olayan & Swarupanandan, 2002; Al-Turki, 2003; Fayed & Al-Zahrani, 2007; Masrahi, Al-Turki & Sayed, 2010; Masrahi, 2012), including the description of *Leptadenia jazanica* as a new species from eastern Tihama and Tallan mountain (Masrahi, 2015). Sand dune habitats represent one of the main aspects of desert environments, especially in the arid belt of the Sahara and Arabian Peninsula. These habitats have a hot, dry climate, with unfavorable features that include a highly permeable substrate with low water holding capacity, high winds with sand accretion, and low and unpredictable rainfall (Novo et al., 2004, 2008; Parsons & Abrahams, 2009). Saudi Arabia occupies the largest portion of the Arabian Peninsula, with a vast area covered by sand dunes; approximately 30% of Saudi Arabia is covered by various sand dunes formed by wind and mobile dunes that provide a unique biological habitat. In Saudi Arabia, inland dunes are essential to the ecosystem (Vincent, 2008; Alatar, El-Sheikh & Thomas, 2012; Alharthi et al., 2020a).

The largest sand dunes habitat in Saudi Arabia is Rub al Khali (the Empty Quarter) in the country’s south and southeastern part, representing the world’s largest continuous sand desert (Kumar & Abdullah, 2011). Dune ecosystems provide highly adapted vegetation because of their low soil water content, high-temperature variation, and unstable soil substrates. One of the primary adaptations of dune plants to moving sand is the rapid elongation of stems and roots in order to keep reproductive and photosynthesis organs above the level of accumulated sand. The elongation of plants typically begins when they are seedlings and continues as they grow (Mandaville, 1998; Vincent, 2008; Alharthi et al., 2020a).

The vegetation of dunes is made up of a series of overlapping plant associations; there are low spots between dunes called swales, which can contain permanent water if groundwater is located very close to the surface (Bowers, 1982; Alatar, El-Sheikh & Thomas, 2012; Alharthi et al., 2020a). Therefore, plant life in the harsh habitats of sand dunes mainly depends on strategies for adjusting to the consequences of water stress (Wainwright, 2009; Remesh, Masrahi & Sayed, 2020; Masrahi, 2020). Although this type of vegetation depends on physical factors—such as the pattern of sediment distribution, the retention capacity of sand and its depth, and the impacts of sand dunes’ water and nutrient distribution—anthropogenic activities such as desert camping and overgrazing contribute to the alteration of their morphology (Bowers, 1982; Vincent, 2008; Valentini et al., 2020; Alharthi et al., 2020b; El-Sakaty et al., 2022). Therefore, land–surface eco morpho-dynamics, also known as land–surface morphology, are determined by geomorphological processes as well as the presence or absence of vegetation (Valentini et al., 2020).

The family Asclepiadaceae, a subfamily of Apocynaceae, has proven to be a valuable source for finding new bioactive compounds, consisting of approximately 250 genera and 2,000 species; among their many pharmaceutical interests, they possess a wide range of biologically active constituents (Endress, 2004; Wiart, 2006). Among these genera is Leptadenia, which consists of about six species: *L. jazanica*Y. L. *ephedriformis*; hastata (Pers.) Decne.; *L. arborea* (Forssk.) *L. Schweinf*; L. *reticulata* (Retz.) Wight and Arn.; *L. pyrotechnica* (Forssk.) Decne. (Deflers, 1896; Grubben & Denton, 2004; Schmelzer, 2008; Masrahi, 2015). *Leptadenia pyrotechnica* (Forssk.) Decne. (syn. *Cynanchum pyrotechnica* Forssk.) is a typical xerophytic desert plant belonging to the Apocynaceae family (Ali, Kausar & Malik, 2001; Boulos, 2009). *Leptadenia pyrotechnica* is a heavily branched shrub reaching 3 m heights; the roots of the shrub reach depths of up to 12 m (Batanouny & Abdel Wahab, 1973). Because of its elongated and extensive system, it is a good soil binder that fixes sand dunes (Qureshi et al., 2012). The shrub branches usually have green to grey–green leaves that tend to fall off early or are totally absent. The shrub has five-fold, short-stalked, greenish-yellow, hermaphrodite flowers with a double inflorescence and a diameter of 2 mm, which stands in axillary small cymose inflorescences. Fruits are 8–11 cm long, narrow, many-seeded, green, and follicular; when ripe, the seeds release pappus-tufted seeds into the wind (Arbonnier, 2009; Idrees et al., 2016). During the months of August and January, the plant blooms and produces fruit. The fruits look like pods and are cooked as vegetables (Verma et al., 2014).

The phytogeographical distribution of *L. pyrotechnica* has been found to be native to the Mediterranean region and semi-arid deserts, such as in African and Asian countries, where sandy and dry conditions prevail. It also grows in Western India’s northern arid sandy Sahel region, the sandy deserts of Punjab, Sindh, and Pakistan (Burkill, 1985; McLaughlin, 2006), and extending eastwards through the Arabian Peninsula (Goyder, 2003; Singh et al., 2012). Traditionally, *L. pyrotechnica* and its parts have been used in ethnomedicine for various purposes in its native countries in Africa and Asia (Verma et al., 2014; El-Fitiany & Khasawneh, 2022).

In Saudi Arabia, the seeds and whole plant extract are used to treat coughs, flu, and induce lactation (Youssef, 2013). *L. pyrotechnica* fiber is used as an antihistaminic and expectorant. It has been proven to have antibacterial activity against *Staphylococcus aureus* and *Bacillus subtilis*, and is used for wound treatment in Yemen (Al-Fatimi et al., 2007). Plant sap is used to treat eczema and diabetes, as well as other skin conditions. To remove thorns, a latex or leaf paste is applied over the injury (Qureshi, 2002; Upadhyay, Roy & Kumar, 2007). In the Sariska region of Rajasthan, a whole plant infusion is mixed with buttermilk and given to patients with uterine prolapse and stomach disorders (Katewa & Galav, 2006; Upadhyay, Singh & Kumar, 2011). It also treats constipation and is commonly considered good for health (Patel, Kanjaria & Patel, 2010). Moreover, it is traditionally used for fever, cough, kidney disorders, stones, and urinary disease in the sudanodeccanian region of central Sahara (Hammiche & Maiza, 2006).

*Leptadenia pyrotechnica* represents one of the widely distributed xerophytic species inhabiting sandy formations in the arid belt of Mauritania in the Arabian Peninsula. However, this wide distribution of *L. pyrotechnica* in such desert habitats is still largely unexplored in morpho-anatomical studies (Migahid, Wahab & Batanouny, 1972). Consequently, this study aims to investigate some morpho-anatomical features to distinguish the habitat effect on *L. pyrotechnica* plants from two sand dune locations: hyper-arid inland sand dunes (the Empty Quarter) and arid coastal sand dunes (Jazan coastal sand dunes).

## Materials and Methods

### Site description

**Figure 1:**
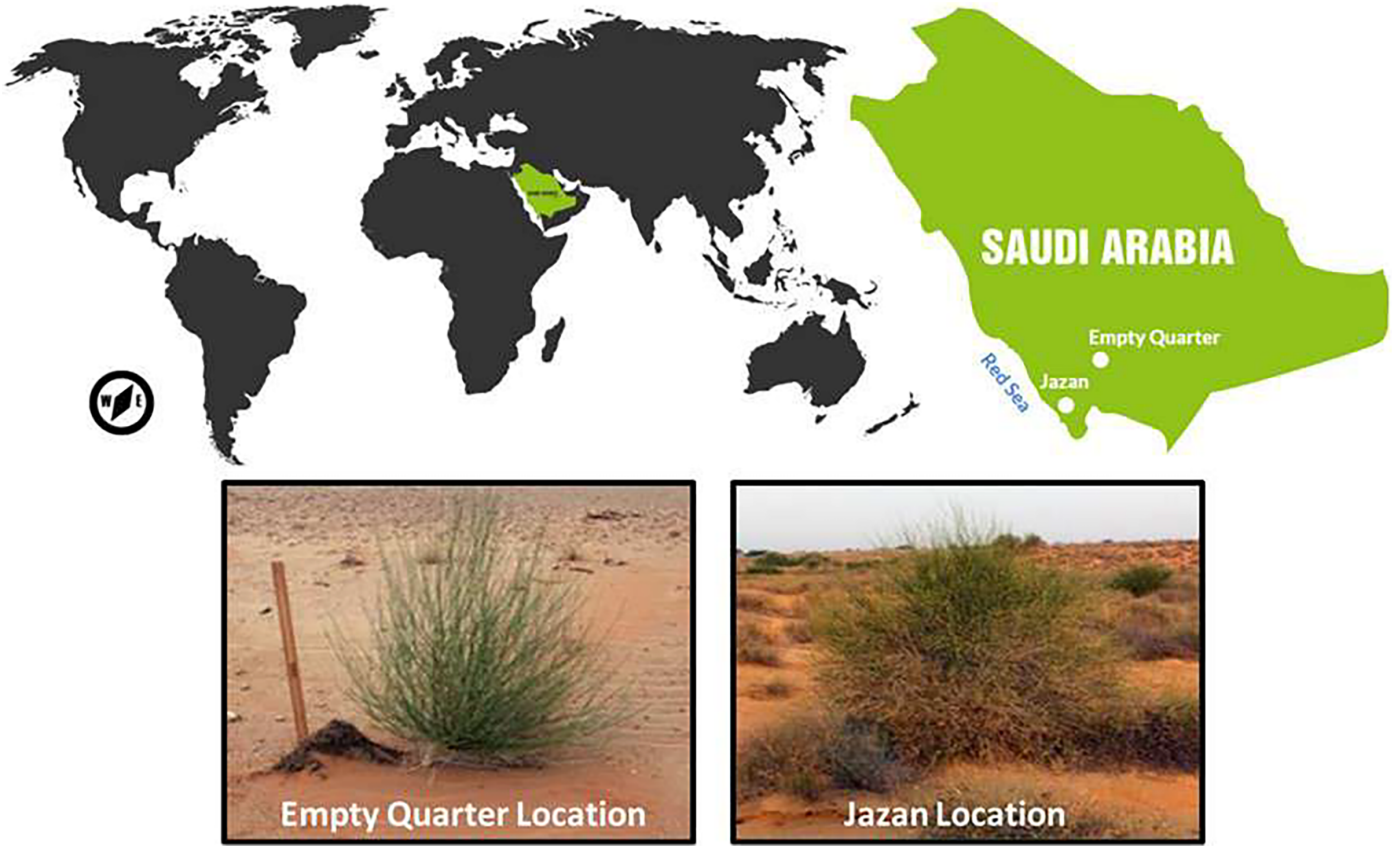
Study areas and sampling localities of *Leptadenia pyrotechnica* in Jazan (coastal sand dune habitat) and Empty Quarter (represented by Oroug Bani M’aradh).

Jazan Region is located in southwestern Saudi Arabia (latitude 17°06′N; longitude 42°33′E) with an average width of about 100 km from east to west (Fig. 1). Generally, the Jazan area experiences hot summers, with mean monthly air temperatures ranging between 30 °C during January and 40 °C during August. Rainfall reaches its maximum (18.0 mm yr^−1^) in December and its minimum (0.8 mm yr^−1^) during June (Masrahi, 2012; Stewart, 2016). The specific sampling point located at the sand dunes near the Red Sea coastal belt of Jazan Province at an altitude 15 m a.s.l.

In south Saudi Arabia, Uruq Bani Ma’arid is a protected area at the western edge of Rub’ al Khali (the Empty Quarter), the world’s largest sand desert. Uruq Bani Ma’arid is situated on the western edge of Rub’ al Khali (the Empty Quarter) in southern Saudi Arabia, known as the world’s largest sandy desert (latitude 19°30′N; longitude 45°30′E), with a total area of 12,658 km^2^. It has a hot and arid climate with infrequent rainfall averaging 30 mm yr^−1^ (Fig. 1). Water drains into the substrate during downpours on the escarpment, where it is retained as underground water (Logothetis et al., 2021). The specific sampling point was located at 19°09′30.7″N and 45°08′50.0″E, at an altitude of ~600 m.a.s.l.

### Collection and identification of plant samples

Six plants belonging to *L. pyrotechnica* were collected during September and October 2021 from both habitats according to Canfield (1941). Identification and taxonomic nomenclature of the plant specimen and chorotypes were evaluated according to the literature (Tackholm & Boulos, 1974; Boulos, 2000). The collected plant materials were washed, dried under shade, and carefully screened to remove any extraneous material, after samples were preserved in 70% alcohol for morpho-anatomical biological studies. Voucher specimens have been deposited in the herbarium of Princess Nourah bint Abdulrahman University (PNUH).

### Soil texture analysis

For the soil texture analysis, a composite sample was obtained by mixing 100 g of three soil samples collected from each location in the field by a sieving method to eradicate any impurities. Soil texture and porosity were determined according to Piper (2019).

### Morphometrical characters

Morphological attributes were investigated for each plant to determine the micro-morphology of epidermal cells and the stomata; this was examined by light microscopy (BA410E, Motic, Kowloon, Hong Kong) connected to a Moticam camera (1080HD, Motic).

Measurements of the morphometrical characters (S/V ratio) and average thickness of the boundary layer (δ^bl^) were taken from fresh cross-sections of the stem, in which the caliper measured the stem’s diameter. The S/V ratio was calculated using the below formula for cylindrical stems according to Mauseth (2000). The S/V ratio for the cylindrical stem = 2/r, where r is the stem radius (mm). The thickness average of the cylinder’s boundary layer (δ^bl^) was calculated using the following formula (Bobich & Nobel, 2001): δ^bl^ (mm) = $5.8{\rm \; }\sqrt {{\rm d}/{\rm v}}$, where d is the cylinder diameter in m and v is the ambient wind speed in m s^−1^ (= 1 m s^−1^ as the average daily wind speed in exposed places of the habitats).

### Anatomical studies

#### Light microscopy

Sections of the stem and roots, including the phloem, xylem, epidermis, cortex, and pith, were stained with safranin and light green, and then examined by light microscopy. Measurements were taken from the LM photomicrograph to estimate the average xylem vessel diameter and frequency. Vessel diameter values were calculated from over 25 determinations (IAWA Committee, 1989). Vessel frequency (VF) represents the number of vessels per 1 mm^2^ of the stem’s cross-sectional area. Vessels with a diameter of fewer than 25 µm were not considered because of their limited contribution to water conductivity (Ewers et al., 1997; Gutierrez, Miguel-Cha & Terrazas, 2009). Vulnerability to cavitation (vulnerability index: VI) was calculated using the equation proposed by Carlquist (1977) as follows: VI = VD/VF, where VD is the vessel diameter (in µm) and VF is the vessel frequency (n/mm^2^).

#### Scanning electron microscope (SEM)

Fresh stem and root samples were divided into 4–6 mm segments, dried and coated by platinum, placed on the double side carbon tape of a 490 aluminum stub, and examined by a field emission SEM (JSM-IT500HRlLA). As in SEM, energy-dispersive X-ray spectroscopy (EDX) involves fixing and dehydrating the sample. Software version 1.8 of NORAN System SIX was used.

### Crystal elements analysis

In order to conduct the elemental analysis of the crystal micropatterns of *L. pyrotechnica*, following Souza, Moscheta & Oliveira (2004), we focused on identifying types of calcium oxalate crystals and their micropatterns, which were processed for scanning electron microscopy and an elemental X-ray analysis for anatomical features and crystal movement. The examination was determined by energy-dispersive X-ray spectroscopy (EDS) (JSM-6380 LA; JEOL, Akishima, Japan).

### Statistical analysis

Macro-, micro-morphology, and anatomy data were subjected to one-way ANOVA followed by Duncan’s *post hoc* test at a significance level of 0.05; they were used to analyze variations in morpho-anatomical characters (Bobich & Nobel, 2001). The statistical analysis was performed using GraphPad Prism Software version 9.1 (San Diego, CA, USA).

## Results

### Soil texture analysis

The results of the four analyzed components of soil texture (*i.e*., coarse sand, fine sand, silt, and clay) of both habitats under study are illustrated in Table 1. Coarse sand was 500 µm, which was lower in the Empty Quarter than in the Jazan coastal sand dunes. Meanwhile, fine sand recorded 250–125 µm, which was higher in the Empty Quarter than in the Jazan coastal sand dunes soil. The percentage of fine soil (<125 µm, including silt and clay) was higher in the Jazan coastal sand dunes soil than in the Empty Quarter. According to the USDA soil texture triangle (Groenendyk et al., 2015), the composition of the soil texture in the Empty Quarter is classified as loamy sand, while the soil texture in the Jazan coastal sand dunes is classified as sandy loam.

**Table 1:** Soil texture analysis from the two habitats, Jazan and the Empty Quarter (EQ).

Habitat	Soil partiale size (µm)			
	Coarse sand	Fine sand	Silt	Clay
	500	250	125	<125
EQ	0.17 ± 0.1	53.17 ± 0.9	32.37 ± 0.42	14.3 ± 0.5
Jazan	13.73 ± 3.25	44.43 ± 3	22.23 ± 4.61	19.6 ± 1.61

### Morpho-anatomical characters

**Figure 2:**
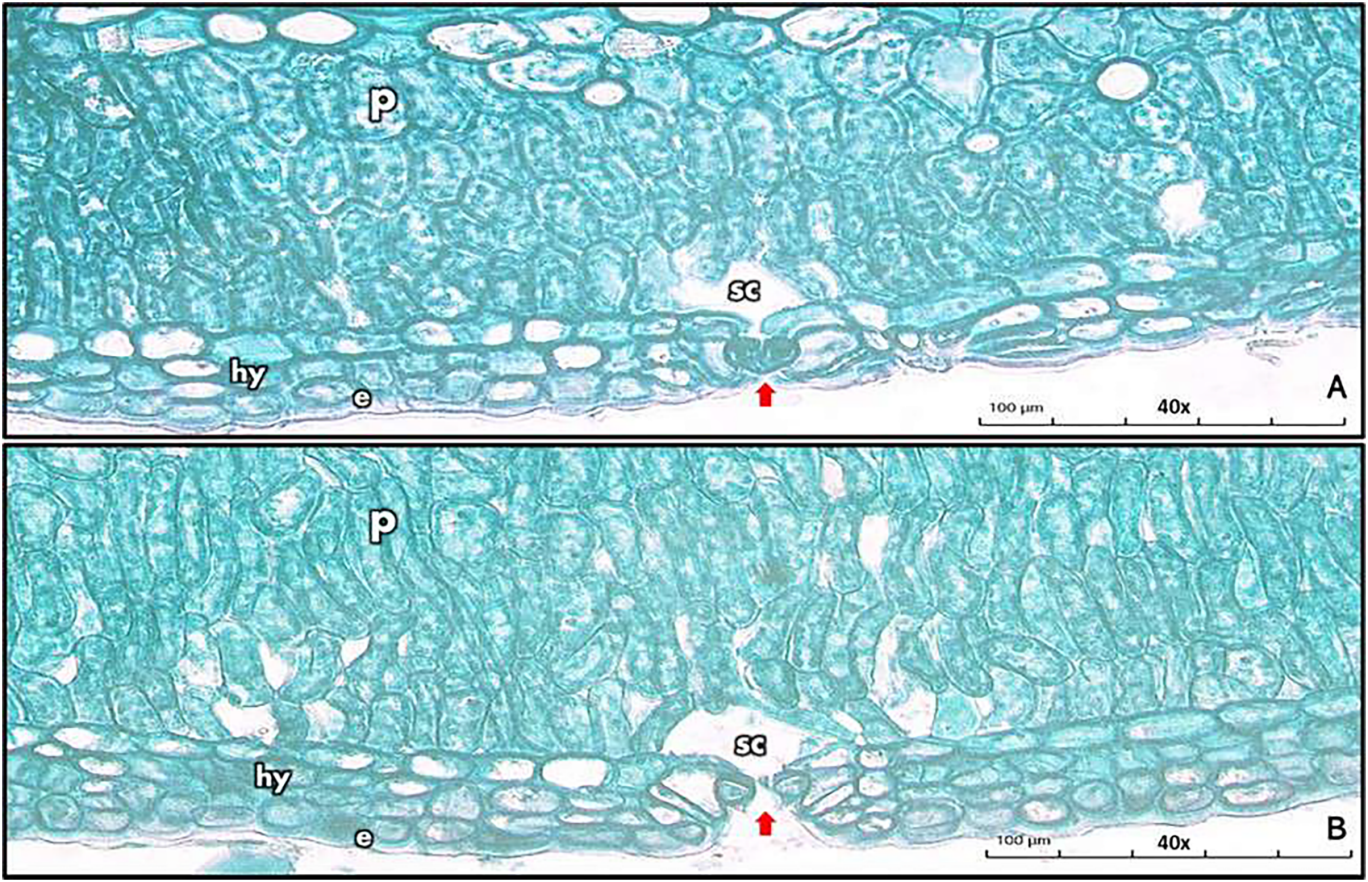
Sunken-type stomata in *L. pyrotechnica* stem from both habitats, the Empty Quarter location (A) and Jazan coastal sand dunes location (B). e, Epidermis; hy, hypodermis; p, palisade cells; sc, substomatal cavity.

The surface-to-volume ratios (S/V) of stems in *L. pyrotechnica* from both habitats were similar, as was the average thickness of the boundary layer δ^bl^ (Table 2). Using a light microscope to examine stomata in the stem revealed a sunken type from both habitats, with more encryption from the Empty Quarter habitat compared to the Jazan coastal sand dunes habitat (Fig. 2).

**Table 2:** Morpho-anatomical characteristics of *Leptadenia pyrotechnica* from Jazan and the Empty Quarter (EQ).

Index	S/V of stem (mm^2^/m)	δ^bl^ of the stem (mm)	VD (µm) of stem and root	VF (N/mm^2^) of stem	VI of stem	PP (Stem and root)	TWT (Stem and root)	VD (µm) of root	VF (N/mm^2^) of root	VI of root
Site										
EQ	1.8 ± 0.5	0.27 ± 0.03	44.2 ± 8.6*	31.0 ± 6.2	1.42	S	Cir	76.9 ± 27.7**	22.7 ± 2.1	3.39
Jazan	1.9 ± 0.3	0.27 ± 0.03	32.7 ± 7.5	43.3 ± 8.1	0.75	S	Cir	57.1 ± 25.1	34.3 ± 11.7	1.66

**Notes:**

S/V ratio of stem, surface- to -volume ratio of Stem; δ^bl^, Boundary layer thickness; VD, Vessel diameter; VF, Vessel frequency; PP, Perforation plates; TWT, Type of wall thickening; VI, Vulnerability index; EQ, Empty Quarter.

* Significance at *P* < 0.01.

** Significance at *P* < 0.0001. S stands for simple, Cir stands for circular.

**Figure 3:**
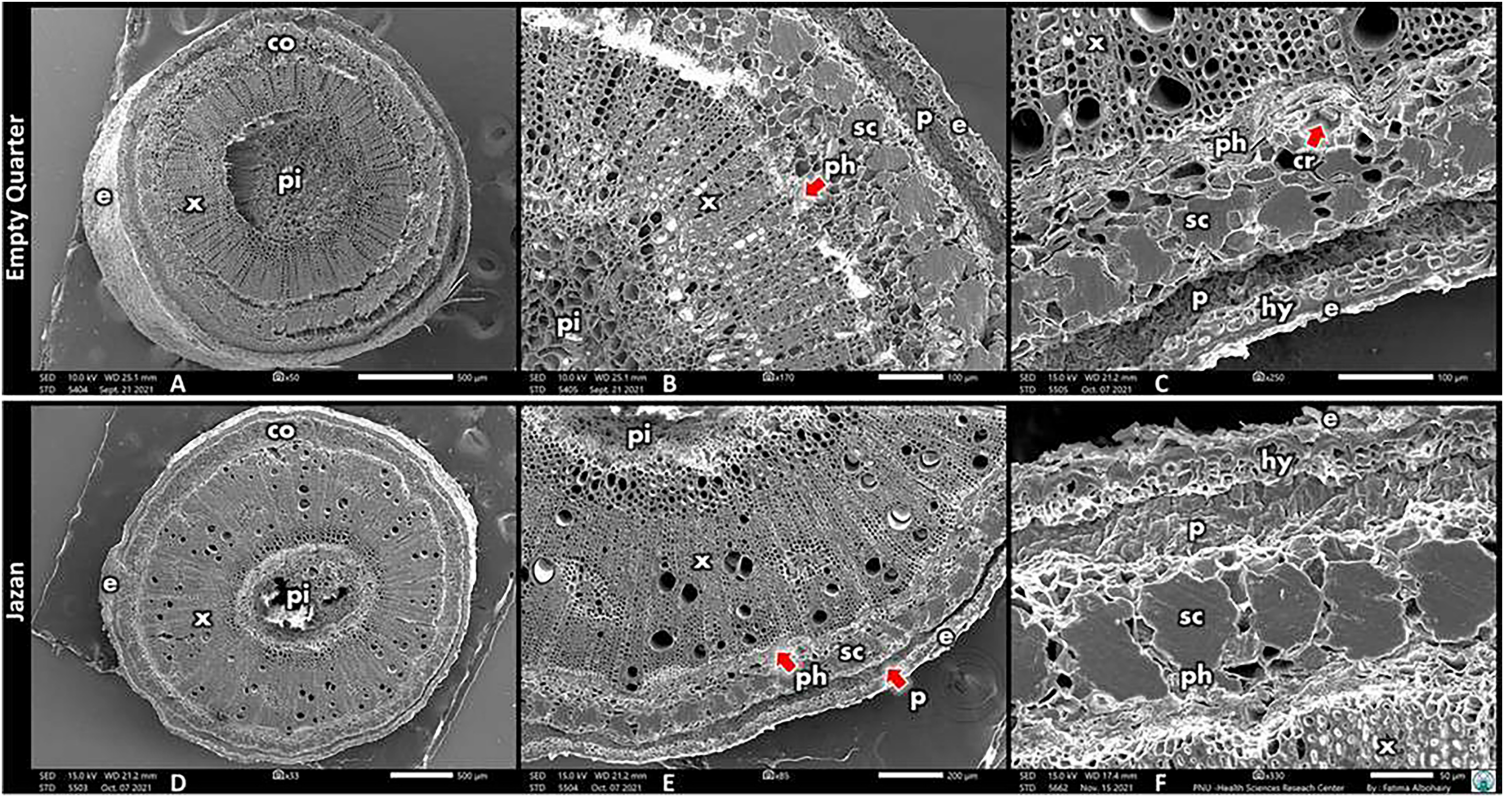
Scanning electron photomicrograph of stem cross-section from both habitats, Empty Quarter location (A–C) and Jazan habitat (D–F). e, Epidermis, hy, hypodermis; co, cortex; p, palisade cells; sc, sclerenchymatous cells; cr, crystals; ph, phloem; pi, pith; x, xylem vessels.

The anatomical structure of *L. pyrotechnica* stems from both habitats is described in Fig. 3. In detail, the stem’s transverse section (TS) revealed a similar pattern, epidermis, and 3–4 layers of the hypodermis. The first layers of the cortex consist of chlorenchyma, in which many layers of palisade cells represent photosynthetic tissue (Figs. 2, 3).

*L. pyrotechnica* in the Empty Quarter had elongated palisade cells compared to the coastal sand dunes of Jazan. The bundles of sclerenchymatous cells (thick-walled, lignified fibers) in the discontinuous cylinder (pericycle) are found beneath chlorenchyma layers. The xylem vessels are scattered throughout the ring of vascular tissue in the transverse section of the stem (Fig. 3). In both habitats, *L. pyrotechnica* showed inner phloem on the side of the medulla. Therefore, the vascular bundle can be classified as the bicollateral vascular bundle. Furthermore, phloem islands (bundles of phloem scattered in the secondary xylem) were observed. In the transverse section (TS), the central part (pith) comprises loose parenchyma cells from both habitats (Fig. 3).

### Root anatomy

**Figure 4:**
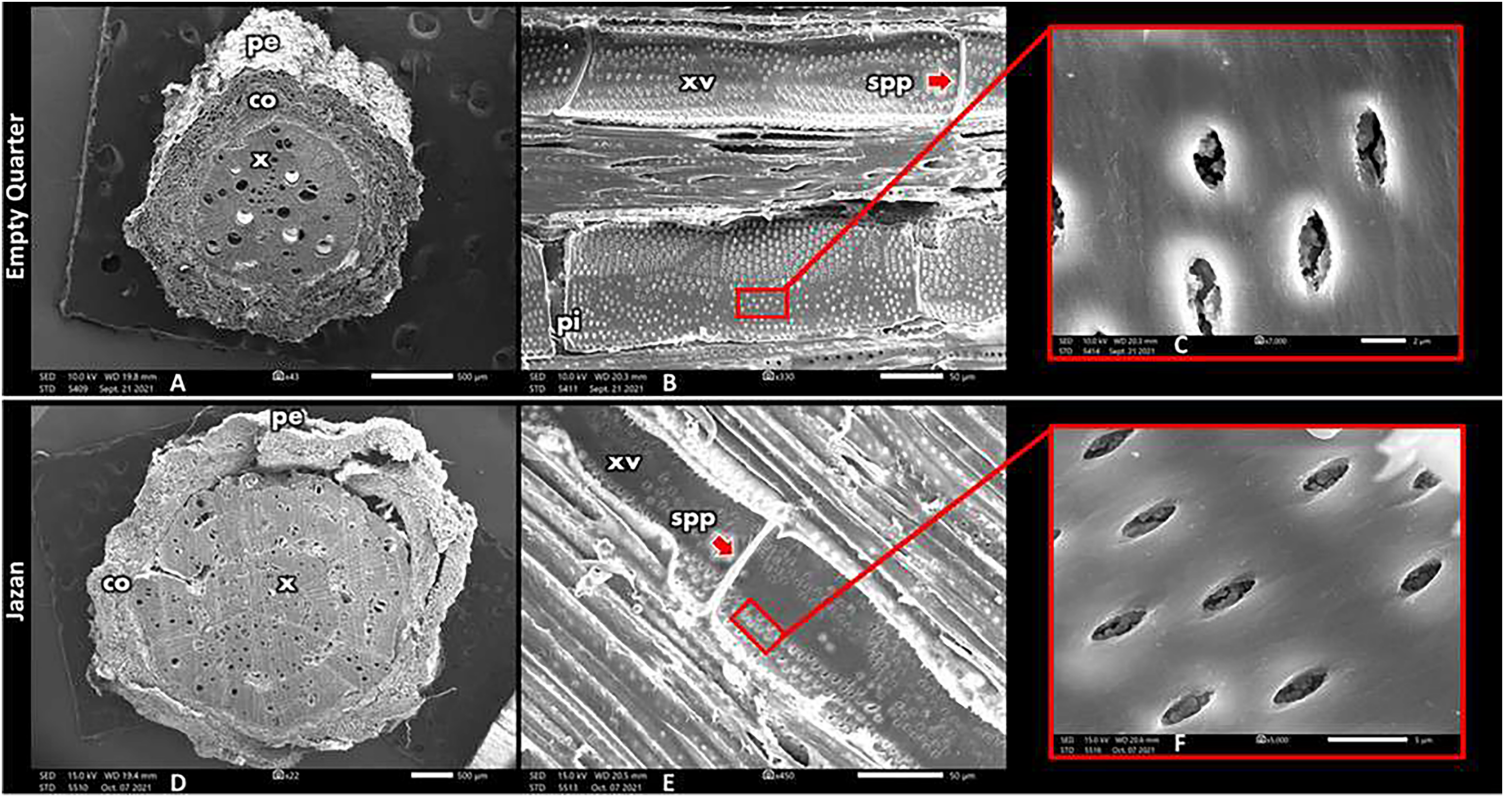
Scanning electron photomicrograph of root cross-sections from both habitats, Empty Quarter location (A–C) and Jazan habitat (D–F). (E and F) Shows zommed vesture pits. pe, Periderm; co, cortex layers; x, xylem; xv, xylem vessel; spp, simple perforation plate.

The root anatomy of *L. pyrotechnica* in both habitats is illustrated in Fig. 4. Periderm layers surround the cortex in the root’s transverse section (TS) (with secondary growth). The vascular cylinder represents the larger area. The vessel diameter was more significant in roots from the Empty Quarter habitat than in roots from the Jazan coastal sand dunes. In contrast, there was less vessel frequency in the roots from the Empty Quarter habitat compared to that from the Jazan coastal sand dunes habitat. As in the stem, these vessel character differences led to a high vulnerability index in root xylem vessels from the Empty Quarter habitat (about double that from the Jazan coastal sand dunes habitat) (Table 2). A longitudinal section (LS) of the root’s xylem vessels showed pits on the inside. Compared with Jazan sand dunes habitat, the root vessels of *L. pyrotechnica* from the Empty Quarter habitat had more pits. Both habitats had vestured pits in root vessels.

### Crystal elements and starch content analysis

**Figure 5:**
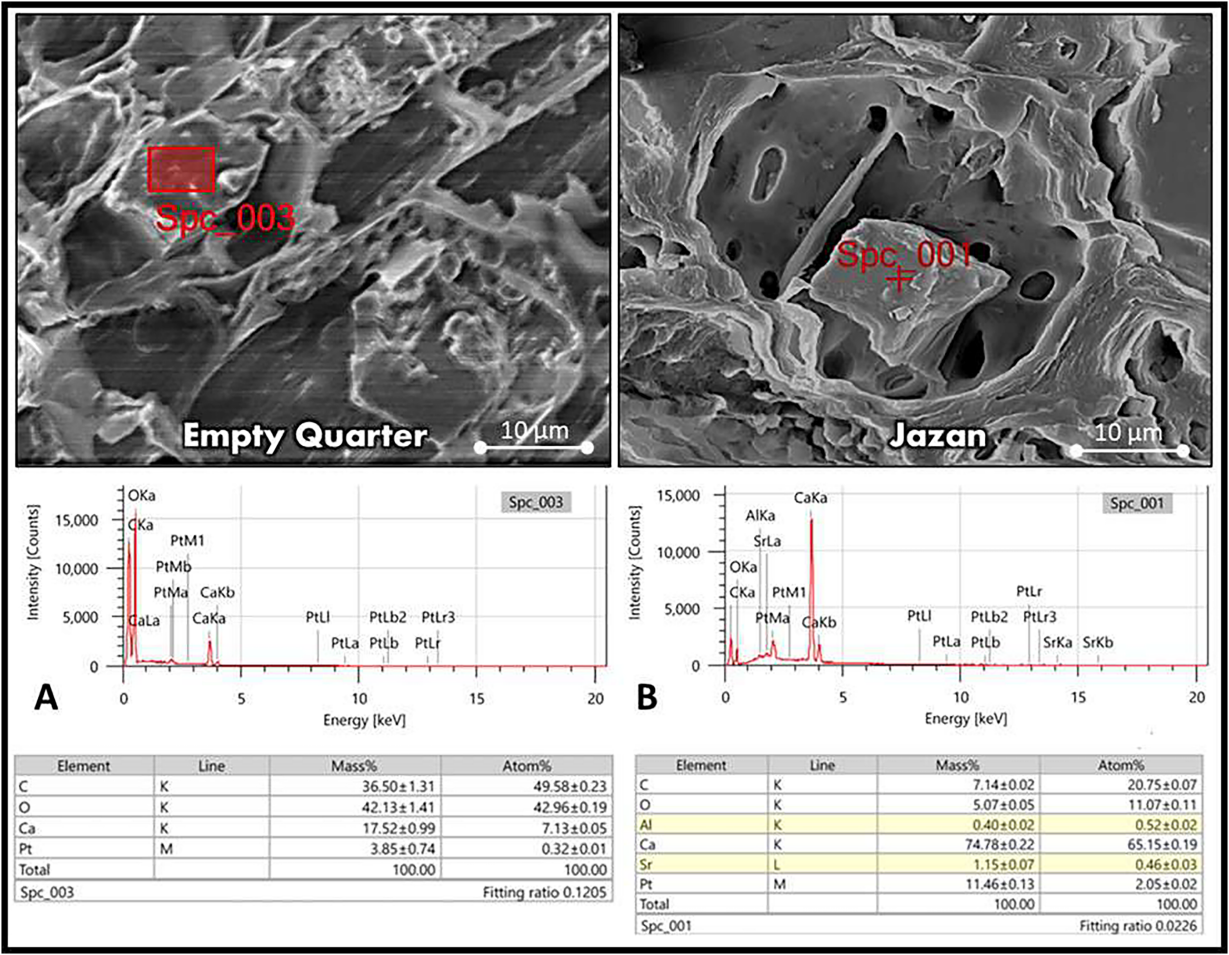
Elemental analysis of *L. pyrotechnica* stem crystals by energy-dispersive X-ray spectroscopy (EDS) from both habitats, Empty Quarter location (A) and Jazan coastal sand dunes location (B).

In both habitats, the last layer of the cortex in the stem was covered with prismatic crystals. More crystals were found in *L. pyrotechnica* from the Jazan sand dunes than in the Empty Quarter pyrotechnica. EDX of the crystals revealed a high concentration of calcium in *L. pyrotechnica* from the Jazan sand dunes, with low carbon and oxygen ratios of 74.78, 7.14, and 5.00 as mass %. At the same time, samples from the Empty Quarter were deficient in calcium concentrations with relatively high carbon and oxygen (17.52%, 36.5%, and 42.13%) (Fig. 5).

**Figure 6:**
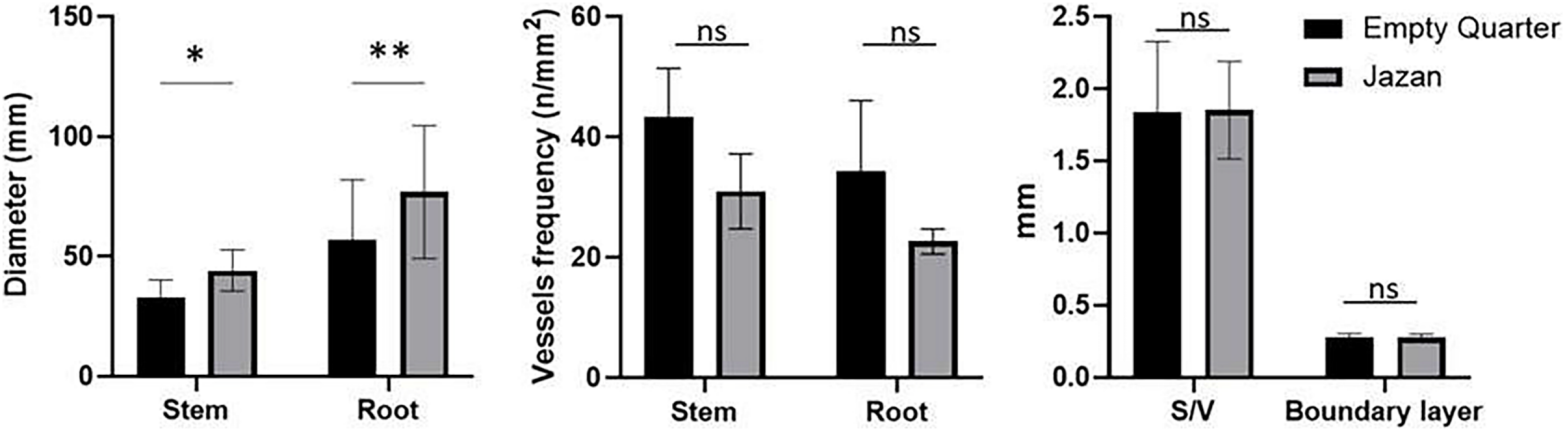
Diameter (mm), vessel frequency (n/mm^2^) of the stem and the roots, S/V and boundary layers *L. pyrotechnica* from Empty Quarter and Jazan. ns, not statistically different; *P < 0.01 and **P < 0.0001.

**Figure 7:**
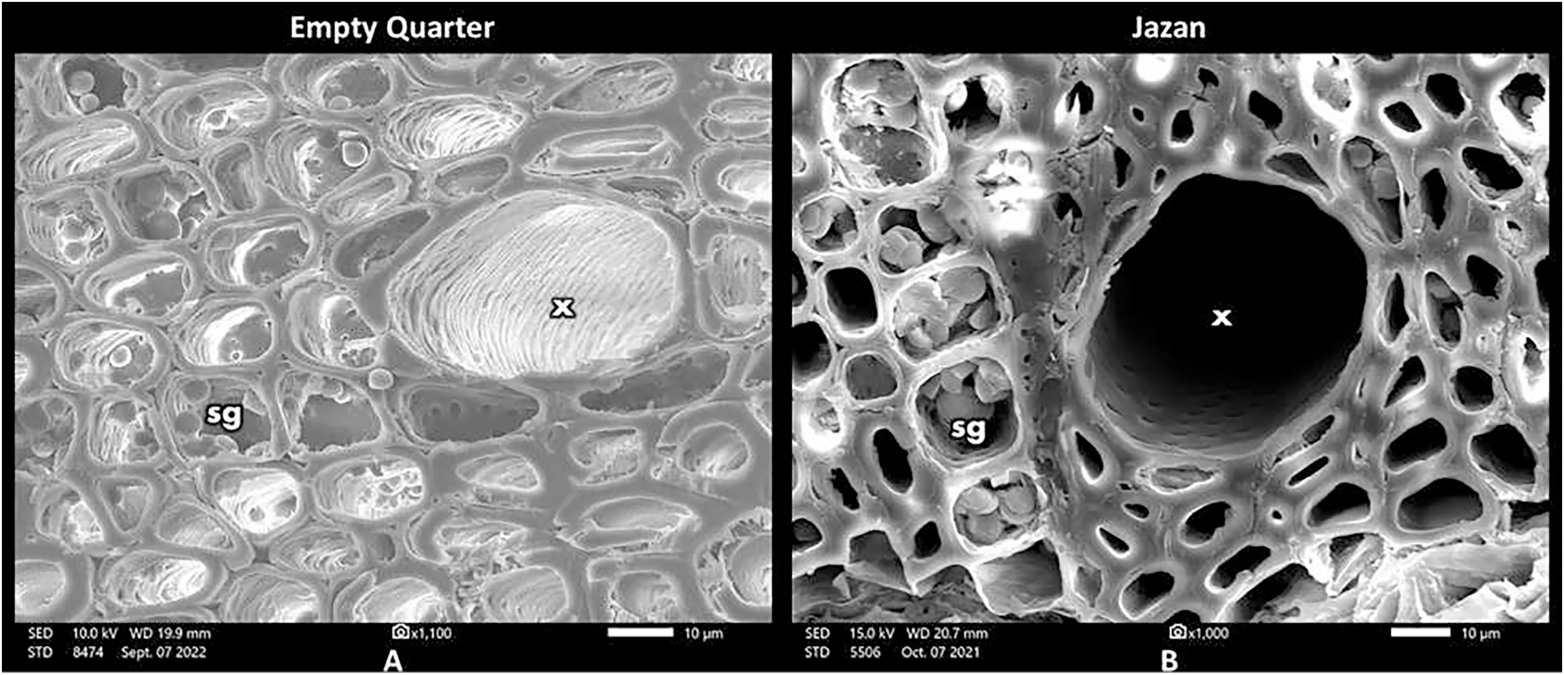
Scanning electron photomicrograph of stem cross-section shows ray parenchyma cells near the vessels with storage of starch grains from Empty Quarter habitat (A) and Jazan coastal sand dunes habitat (B). x, xylem vessel; sg, starch grains.

In the Empty Quarter habitat, vessel diameters were more significant than those in the Jazan coastal sand dunes (Fig. 6). In contrast, vessel frequency was lower in the Empty Quarter habitat stem than in the Jazan coastal sand dunes habitat stem (Fig. 6). Consequently, stem xylem vessels from the Empty Quarter habitat had a high vulnerability index (double that of the Jazan sand dunes habitat). In addition, the type of wall thickening of xylem vessels in *L. pyrotechnica* stem from both habitats was circular (Table 2), and plants from both habitats stored starch grains in ray parenchyma cells near the vessels (Fig. 7).

## Discussion

The Empty Quarter and the Jazan coastal sand dunes are in a hyper-arid zone and are mainly composed of xeromorphic shrubs and herbs. In arid habitats of the Sahara and Arabian Peninsula, *Leptadenia pyrotechnica* represents one of the most drought-resistant plants (Migahid, Wahab & Batanouny, 1972; Goyder, 2003; Singh et al., 2012). Plant life in arid habitats largely depends on multiple adaptation strategies to cope with water scarcity and high evaporation rates (Wainwright, 2009; Remesh, Masrahi & Sayed, 2020). It has been suggested that morpho-anatomical characteristics represent leading strategies in xerophytic species (Batanouny, 2001; Masrahi, 2020). Plant stems in both habitats (the Empty Quarter and the Jazan coastal sand dunes) have the same low S/V ratio, indicating a high capacity to store water, with a low surface area that reduces water loss by transpiration (Gibson, 1996; Mauseth, 2000). The boundary layer (δ^bl^) is a significantly related feature to shape the S/V ratio (Masrahi, 2020). Because of the thin boundary layer of *L. pyrotechnica* stems, the temperature can be maintained at an optimum level without transpiration cooling (Gibson, 1996). Additionally, the cylindrical stem does not intercept direct solar irradiance nor a leaf shape, as only half of the stem is exposed at any given time (Gibson, 1998).

*Leptadenia pyrotechnica* from the Empty Quarter had more encrypted stomata than from the Jazan coastal sand dunes; it has been suggested that sunken stomata reduce stomatal conductance, which reduces transpiration (Mauseth, 1988; Roth-Nebelsick, 2007; Masrahi, 2020). These features of sunken stomata compensate (micro-climatically) the thin boundary layer of the whole stem, greatly enhancing water use efficiency (Masrahi, 2020). *Leptadenia pyrotechnica* revealed an epidermis with many layers of hypodermis in both habitats; this feature of xerophytes increases cuticular resistance to water loss, reducing the transpirational rate (Fahn & Cutler, 1992). Plants from the Empty Quarter have palisade cells in their photosynthetic tissue, which are more elongated; there is evidence that more elongated palisade cells allow for more efficient light distribution, allowing chloroplasts to relocate to a larger surface area and increase their ability to absorb CO_2_ (Matthes et al., 2022). In addition to chlorenchyma layers, sclerenchymatous cells surround vascular tissue, contributing to stem strength (Crang, Lyons-Sobaski & Wise, 2018). Additionally, these bundles of sclerenchymatous cells reduce palatability, thereby decreasing herbivorous grazing (Y. Masrahi, 2022, self-communication). *L. pyrotechnica* stems from both habitats showed prismatic crystals in the last layers of the cortex, close to sclerenchymatous cells and vascular bundles. Calcium oxalate crystals were reported to be present in many Apocynaceae genera, including Leptadenia (Denni, Mammen & Ramesh, 2012; Tütüncü Konyar, Öztürk & Dane, 2014; Naik, Harisha & Acharya, 2018).

*Leptadenia pyrotechnica* from Jazan contained more calcium oxalate crystals than the Empty Quarter’s. Aridity conditions and soil Ca levels influence the formation of calcium oxalate crystals in some plants (Brown, Warwick & Prychid, 2013). Additionally, calcium oxalate crystals may protect plants from herbivores (Peschiutta et al., 2020). Previous studies found that herbivorous plants had higher crystal densities than plants not subjected to herbivory (Molano-Flores, 2001). The quantity of Ca, C, and O (%) in calcium oxalate crystals revealed variable levels of these elements between stems from both habitats. In the Empty Quarter, the stem of *L. pyrotechnica* showed low Ca levels and relatively high C and O levels.

In contrast, the *L. pyrotechnica* stem from the Jazan coastal sand dunes revealed high Ca levels with low C and O levels. It has been suggested that calcium oxalate crystals represent a vital storage reservoir of Ca, which regulates these element levels (López-Macías et al., 2019). High levels of Ca in the crystals may be related to the inability to mobilize Ca and/or protection from herbivory (Molano-Flores, 2001; Delian et al., 2014; López-Macías et al., 2019). In the transverse section (TS) of the stem from both habitats, the ring of vascular tissue represents the larger area with scattered xylem vessels. Vessel diameter and frequency are the most critical hydraulic traits in the xylem for coping with drought conditions (Lindorf, 1994; Masrahi, 2014). Embolization safety is directly related to the values of these traits (Carlquist, 1977; Yang et al., 2008).

Compared to the Jazan coastal sand dunes habitat, the Empty Quarter habitat vessels had a larger mean diameter in the stem, although the vessel frequency was lower. Therefore, stem xylem vessels in the Empty Quarter habitat had a vulnerability index (VI) that was twice that of those in the Jazan coastal sand dunes. Contrary to this, stem vessels from the Jazan coastal sand dunes habitat were more embolism resistant. As xylem conduits in stems are continuous paths from roots, hydraulic traits are nearly identical. In contrast, wide vessels (large diameter) have high hydraulic conductivity (Masrahi, 2014). Relatively more comprehensive vessels in the stem of *L. pyrotechnica* from the Empty Quarter may relate to edaphic characters, in which soil is classified as loamy sand (coarse texture, larger pores), having low water holding capacity with more free water movement between soil pores. In contrast, the Jazan coastal sand dunes habitat has more fine soil (sandy-loam, meso-, and micropores) and has a higher water holding capacity with less free water movement between soil particle pores (Beaudette, Roudier & O’Geen, 2013).

Starch grains were observed in the ray parenchyma of stems from both habitats. It was reported that starch grains, besides supporting growth, may enhance water flow by hydrolysis into soluble sugars, which enter the vessels and reduce the solute potential of sap, keeping the gradient flow of water in a continuum soil–root–stem and reducing the risk of embolism (Carlquist, 1977; Carlquist & Schneider, 2001; Masrahi, 2014). Phloem in the transverse section (TS) of the stem was at both sides of the xylem (bicollateral bundles), and was present as scattered “islands” in the secondary xylem (phloem islands). This arrangement of phloem keeps it in proximity with xylem conduits. It has been reported that phloem may export some signal for starch hydrolysis, which would drive sugar efflux into embolized conduits with consequent osmotic water flows and refilling (Salleo, Trifilò & Lo Gullo, 2006). When phloem distribution matches the xylem vessel characteristics mentioned above, water transport and drought resistance are enhanced in arid habitats. According to both habitats, *L. pyrotechnica*’s root transverse section primarily consists of the vascular cylinder; besides supporting the root system, this feature supports and enhances water uptake and transport. It was found that the xylem vessel of the Empty Quarter habitat had similar hydraulic characteristics (diameter and frequency) to the stem, resulting in a high vulnerability index (VI) in the root xylem vessel compared to the stem (which was higher in the root compared to the stem). According to the above, plant life may be supported by these VI values under prevailing conditions of water stress because of their edaphic characteristics. In contrast, vestured bordered pits were observed in the vessel wall of vessels from both habitats. This type of pit, which represents bordered pits with protuberances from the secondary cell wall of the pit chamber, is suggested to increase hydraulic resistance, help in embolism repair, and promote safer water transport (Jansen et al., 2004; Carlquist & Raven, 2018). Roots from the Jazan coastal sand dunes habitat have fewer vestured pits than those from the Empty Quarter habitat. In *L. pyrotechnica* from the extremely arid Empty Quarter habitat, having more vestured pits could compensate for a high VI value and support safe water transport from the root to the stem. However, soil texture in both habitats was generally sandy; as in most sand dune habitats, the Empty Quarter soil was coarser in texture (loamy sand) compared with a less coarse texture (sandy loam) in the case of the Jazan coastal sand dunes. As discussed above, the Empty Quarter soil has a low water holding capacity with more free water movement between soil pores, while Jazan coastal sand dunes soil has a higher water holding capacity with less free water movement between soil particle pores. These soil traits reflect the distribution and density of *L. pyrotechnica* and other vegetation components in both habitats.

## Conclusions

This study describes *Leptadenia pyrotechnica* as one of the prevailing xerophytic shrubs in the wide range of arid sand dune habitats in Saudi Arabia. It includes information about the background of the arid habitat of the Jazan coastal sand dunes and the hyper-arid habitat of the Empty Quarter. Most morpho-anatomical traits of *L. pyrotechnica* were similar in stems and roots. Sclerenchymatous cells were noted in both habitats, tangled around vascular tissues and forming bundles of sclerenchymatous cells between xylem conduits in both stems with low S/V ratios. High evaporation rates and low precipitation seem to be general adaptive strategies for stems. Meanwhile, *L. pyrotechnica* from the Empty Quarter’s hyper-arid habitat showed more encrypted stomata, longer palisades cells, less calcium oxalate crystals with low calcium content, and a high xylem vulnerability index in comparison to the same traits of *L. pyrotechnica* from the Jazan coastal sand dunes. Moreover, differences were observed in specific anatomical characteristics, especially in xylem vessel characteristics. The Jazan coastal sand dunes habitat had a higher vulnerability index than the Empty Quarter habitat. Additionally, vestured bordered pits were more abundant in the Empty Quarter habitat than in the Jazan coastal sand dunes habitat. These differences in specific anatomical characters may be largely correlated with prevailing climatic-soil conditions, in which the Empty Quarter habitat is characterized by more drought with a more coarse textured soil (loamy sand). In contrast, the Jazan coastal sand dunes habitat is characterized by fewer drought conditions with a relatively less coarse textured soil (sandy loam). In conclusion, the morpho-anatomy of *L. pyrotechnica* from both habitats represents effective adaptation strategies against highly stressful conditions, with anatomical traits correlated to each habitat in accordance with climatic and soil conditions.

## Supplemental Information

10.7717/peerj.15320/supp-1 Supplemental Information 1 Raw Data.

## Additional Information and Declarations

### Data Availability

The following information was supplied regarding data availability: The raw data are available in the Supplemental Files.
